# Erythrocyte and Serum Folate Collection Techniques: A Multi-Method Study of Folate Status

**Published:** 2020-02-10

**Authors:** J Croff, C Tan, AL Chiaf, ML Hartwell, EK Crockett, TK Teague

**Affiliations:** 1 Department of Rural Health, Oklahoma State University Center for Health Sciences, Tulsa, USA; 2 Integrative Immunology Center, University of Oklahoma-Tulsa, Tulsa, USA; 3 Chiaf’s Research, School of Counseling, Counseling Psychology and Community Health Sciences, College of Education, Health and Aviation, Oklahoma State University, Stillwater, USA; 4 Department of Surgery and Psychiatry, University of Oklahoma School of Community Medicine, Tulsa, USA; 5 Department of Pharmaceutical Sciences, University of Oklahoma College of Pharmacy, Tulsa, USA; 6 Department of Biochemistry and Microbiology, Oklahoma State University Center for Health Sciences, Tulsa, USA

**Keywords:** Folate, Serum, Erythrocyte, Linear flow chromatography, Arrayit card dried blood spots, Whatman paper dried blood spots, Venous blood

## Abstract

**Introduction:**

Public health programs aimed at identifying and monitoring individuals at risk of specific nutrient deficiencies may benefit from advances in biospecimen sampling techniques that allow for easier in-the-field collections. Such advances may be particularly important for those of childbearing potential, in order to identify individuals at risk of low folate status due to sub-optimal nutriture. Folate is a critical nutrient of interest among women of childbearing potential because suboptimal levels are a primary contributor to neural tube defects. Whatman Paper Dried Blood Spots (WDBS) are a convenient method for assessing folate; however, a major drawback of WDBS has been the inability to separate serum from erythrocyte folate in these samples.

**Aim of study:**

The purpose of this study was to test the feasibility of using newer linear flow chromatography DBS cards to measure serum and erythrocyte folate.

**Method:**

A convenience sample (n=27) was recruited to assess folate values collected by venous blood draw, Whatman paper, and linear flow chromatography cards. These sampling techniques allowed for assessment of erythrocyte and serum folate values collected via different methods. Folate levels in the samples were assessed using standard *Lactobacillus casei* microbiological assays.

**Results:**

Erythrocyte folate values from the two blood spot methodologies (Whatman paper and linear flow chromatography DBS) indicate a strong linear relationship, with 92% of variance accounted for in a linear regression analysis. Similarly, venous blood samples and linear flow chromatography DBS values accounted for 88% of the variance.

**Conclusion:**

Linear flow chromatography dried blood spot cards are useful for assessment of erythrocyte and serum folate. Values had a strong, positive, linear relationship to serum and erythrocyte folate values from other validated methodologies, including Whatman dried blood spots and venous whole blood samples.

## Introduction

Inadequate folate status is the primary contributor of spina bifida, anencephaly, and other Neural Tube Defects (NTDs) [1–3]. Globally, annual prevalence of NTDs exceeds 300,000 and neonatal deaths exceed 40,000 [4]. In developing countries, 17–70% of neonatal deaths from birth defect are attributable to NTDs [5]. In the U.S, NTD prevalence is 5.4 per 10,000 pregnancies [6]. Poor folate status in women of childbearing potential is also associated with several other pregnancy-related problems, including abruptio placentae; preeclampsia, spontaneous abortion, stillbirth, preterm delivery, and low birth weight [7–15]. Recent studies have identified connections between higher folate levels during pregnancy and reduced risk of autism spectrum disorders and better cognitive functioning scores when children are 9 and 10 [16,17].

One common methodology for assessing folate status is use of Dried Blood Spots (DBS). DBS can be collected by non-medically trained personnel in non-medical environments. Moreover, the low blood volume requirement and ease of procurement improve participation in research [18]. Finally, and critically to public health research and intervention work, the use of dried blood spots reduces exposure to biohazards [19]. Dried blood spots may be particularly useful when the research of interest focuses on a small critical group of biomarkers or nutrients, like folate status. One major weakness of previous DBS methodology is that it did not allow the examination of folate in serum. However, new DBS card technology includes linear flow chromatography which allows analysis of both RBC and serum in the same DBS. In order to explore the use of this new technology for folate analysis, we are comparing linear chromatography to traditional methods as described in detail in the methods section.

## Materials and Methods

**Figure 1:**
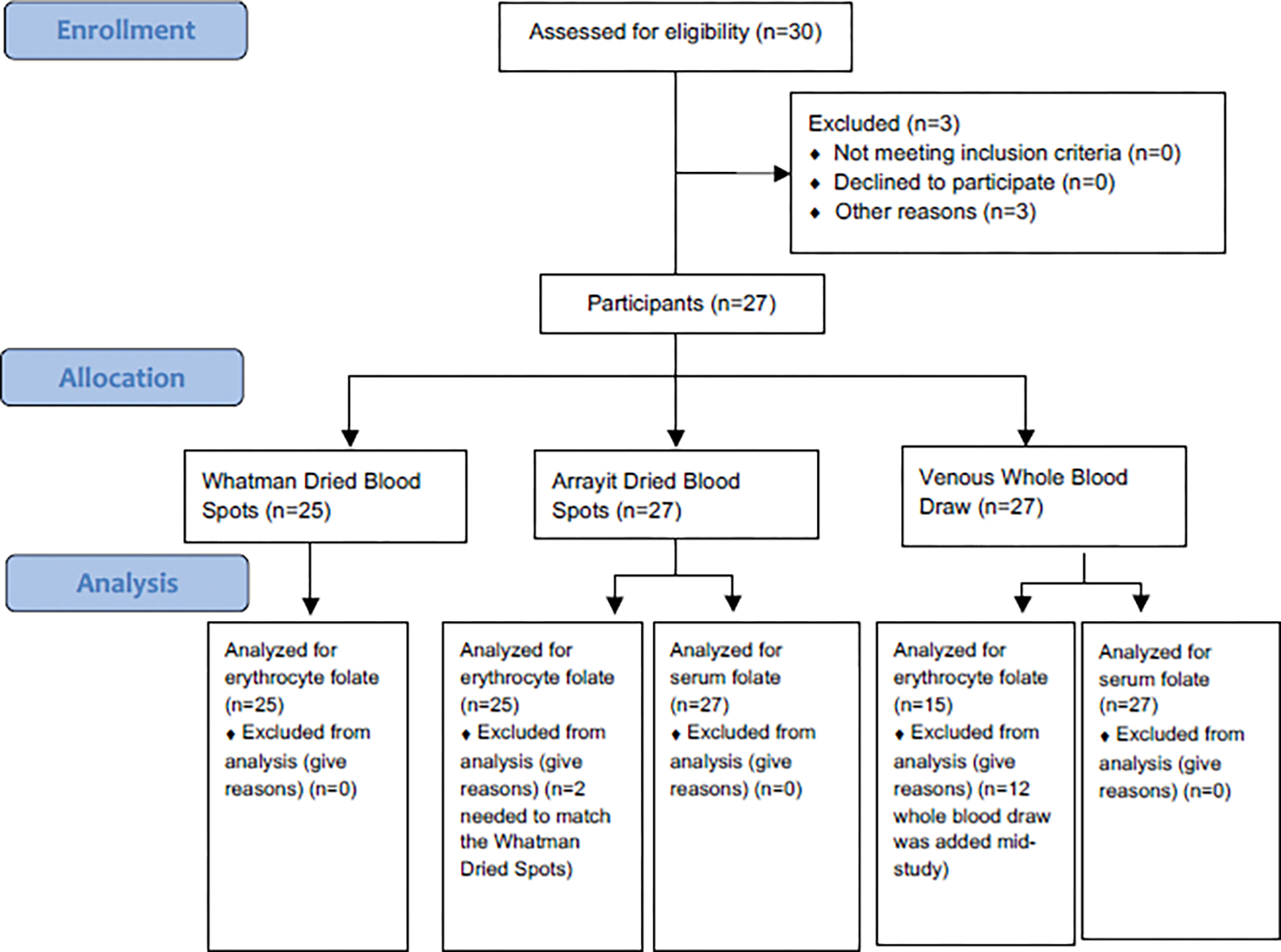
Consort flow diagram. **Note:** ^1^Arrayit Dried Blood Spots; ^2^Whatman Dried Blood Spots

A convenience sample of 27 participants were recruited for this study. Participants were recruited by use of flyers on an urban academic medical campus in Tulsa, OK. The only criteria being age of participants between 18 to 75 years (mean=37.52; SD=15.44). The majority of participants (81.5%, n=22) were female. There were no inclusion/exclusion criteria for the study. All participants were consented by laboratory staff. This study compares the folate values of DBS RBC using linear chromatography cards, DBS RBC using traditional Whatman paper, and whole blood RBC collected with Vacutainer EDTA tubes. Additionally, serum folate values from linear chromatography card DBS were compared to folate values in serum collected using standard blood tubes. Comparisons were made between two blood sampling techniques (finger stick and venous blood draw) to be used across three methodologies:Whatman paper dried blood spots; Arrayit dried blood spots, and venous whole blood. Figure 1 shows the flow of participants in this study from enrollment to analysis.

### Whatman paper Dried Blood Spots (WDBS)

Finger sticks were used to drop blood onto Whatman 903 Proteinsaver Cards (GE Healthcare). This dried blood spot methodology has been previously validated for measurements of erythrocyte folate [20,21]. A serious limitation of WDBS is that they cannot be used to assess serum folate or other serum factors that require separation from red blood cells and/or leukocytes [22]. Multiple 3 mm punches from each WDBS were stored at −80°C until analysis. One 3 mm punch from each WDBS was used for folate and hemoglobin analyses. Each 3 mm punch was assumed to contain 2.4 uL of whole blood.

### Arrayit Dried Blood Spots (ADBS)

Finger sticks were used to drop blood onto Arrayit blood cards (Arrayit Corporation, Sunnyvale, CA), which use linear flow chromatography to separate whole blood serum from erythrocytes and leukocytes. After serum separation, four 3-mm punches from the serum area on each card were used for serum analysis and one 3-mm punch from the center of the RBC area on each card was used for RBC folate and hemoglobin analyses. Punches were stored at −80°C until analysis. According to the manufacturer, each 3-mm punch should contain a liquid volume of 1.4 uL, which was the value used for folate concentration calculations.

### Venous whole blood and serum samples

Venous blood is the gold standard for assessing erythrocyte and serum folate values. Blood was obtained by venipuncture and collected into BD Vacutainer tubes (Becton Dickinson). For Venous Whole Blood (VWB) assessments, blood was collected into BD Vacutainer K2 EDTA tubes and diluted 1/11 in AATX buffer (5g ascorbic acid/L containing 0.1% by volume Triton X-100) to generate lysates as previously described [20]. The lysates were immediately frozen in order to keep the folate in a reduced state and then stored at −80°C until hemoglobin and folate analysis [23]. For serum assessments, blood was collected into BD Vacutainer serum tubes and centrifuged at 1,300 g for 10 minutes at room temperature. The serum fractions were stored at −80°C until folate analysis.

### Folate analysis

Regardless of sampling technique, *Lactobacillus casei* microbiological assays of folic acid derivatives were used to assess erythrocyte or serum folate status, as appropriate. Folate assays were performed using ID-Vit^®^ Folic Acid Assay kits according to the manufacturer’s instructions. Serum was extracted from the ADBS samples by adding 200 uL of ID-Vit^®^ Folic Acid Assay sample preparation buffer to the four punches from each card. Blood components from the WDBS and ADBS RBC punches were extracted by adding 75 uL AATX buffer to each punch. The punches were then shaken at 300 rpm in the presence of extraction buffer for 1.5 hours at room temperature. Eluates from the whole blood lysates, WDBS punches, and ADBS RBC punches were also evaluated for hemoglobin concentrations using a standard hemoglobin assay kit from Sigma-Aldrich (St. Louis, MO). Pooled serum was included in each plate to serve as identical controls across all the assays. The microbiological plates were incubated at 37°C for 48 h and then turbidity read at 620 nm using a Bio-Rad microplate reader. ADBS RBC folate results were given as Hemoglobin-Folate (HF) values using the following formula for calculation: RBC Hemoglobin folate (pmol/g)=RBC folate (pmol/L)/hemoglobin (g/L). For WDBS and whole blood RBC folate we used the following formula to correct the serum folate level and hematocrit (HCT):RBC folate (pmol/L)=(WDBS or whole blood lysate folate)-serum folate × (1-HCT))/HCT where HCT was assumed to be 0.4 as previously described [23].

### Statistical methods

Regression analyses were conducted on samples from the same participant to assess the linear relationship between erythrocyte folate values from the two blood spot methodologies and vacutainer blood samples and linear flow chromatography DBS serum folate. All subjects gave their informed consent for inclusion before they participated in the study. The procedures followed were in accordance with the ethical standards of the institution on human experimentation and approval was obtained from the Institutional review board regarding human subjects.

## Results

### Arrayit Dried Blood Spots (ADBS) *vs.* Whatman Dried Blood Spots (WDBS)

**Figure 2:**
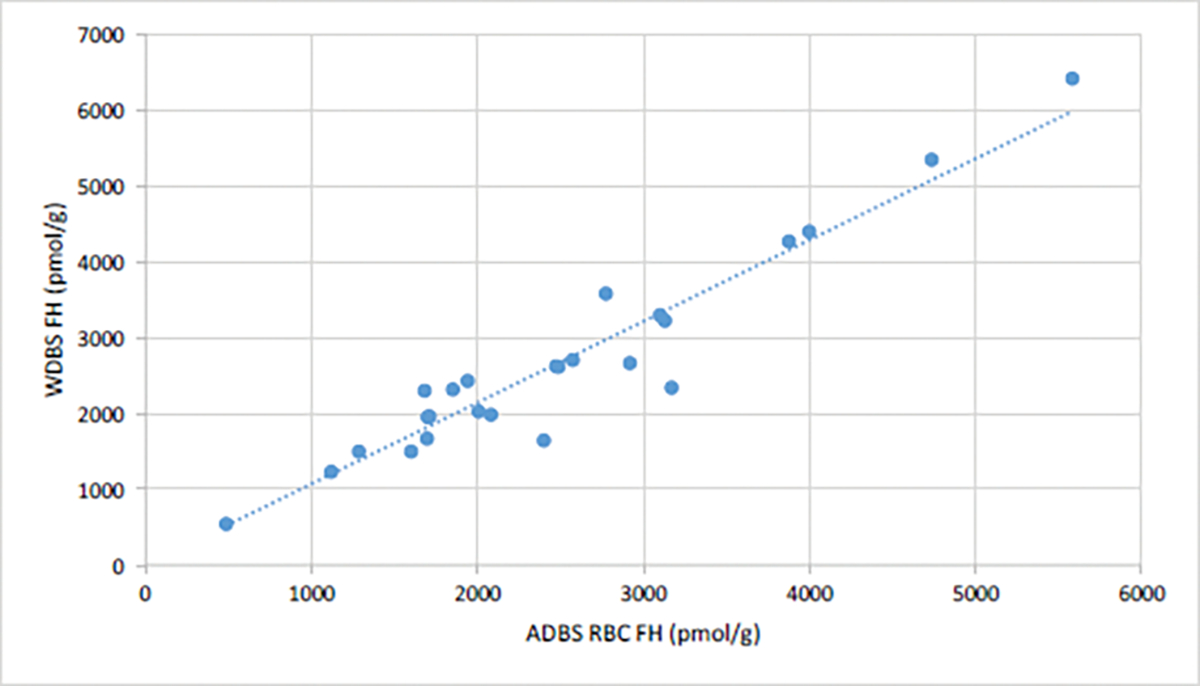
Erythrocyte folate values ADBS^1^ and WDBS^2^. **Note:** ^1^Arrayit Dried Blood Spots; ^2^Venous Whole Blood Sample.

In order to test the utility of using serum separator cards for erythrocyte folate measurements, we first compared erythrocyte folate levels in blood samples collected using Arrayit Dried Blood Spots (ADBS) to those collected using standard WDBS. Twenty-five subjects gave both DBS samples: they were 84.0% female (n=21), and the mean age of participants was 36.84 (SD=15.54, range=22–65). Erythrocyte folate values from the two methodologies indicate a strong linear relationship with regression accounting for most variance (R^2^=0.920 (Figure 2). A linear regression analysis suggests that WDBS=1.0726 × ADBS.

### Arrayit Dried Blood Spots (ADBS) *vs.* Venous Whole Blood (VWB)

**Figure 3:**
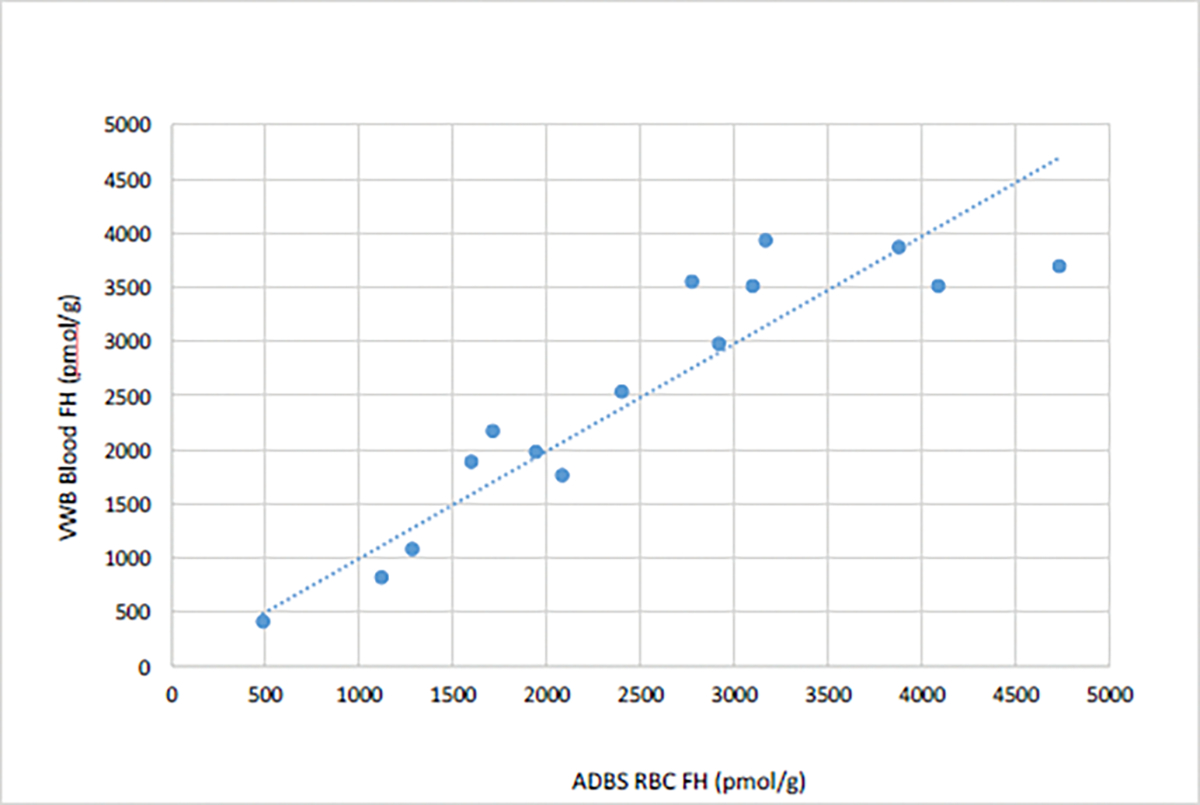
Erythrocyte folate values ADBS^1^ and VWBS^2^. **Note:** ^1^Arrayit Dried Blood Spots; ^2^Venous Whole Blood Sample.

**Figure 4:**
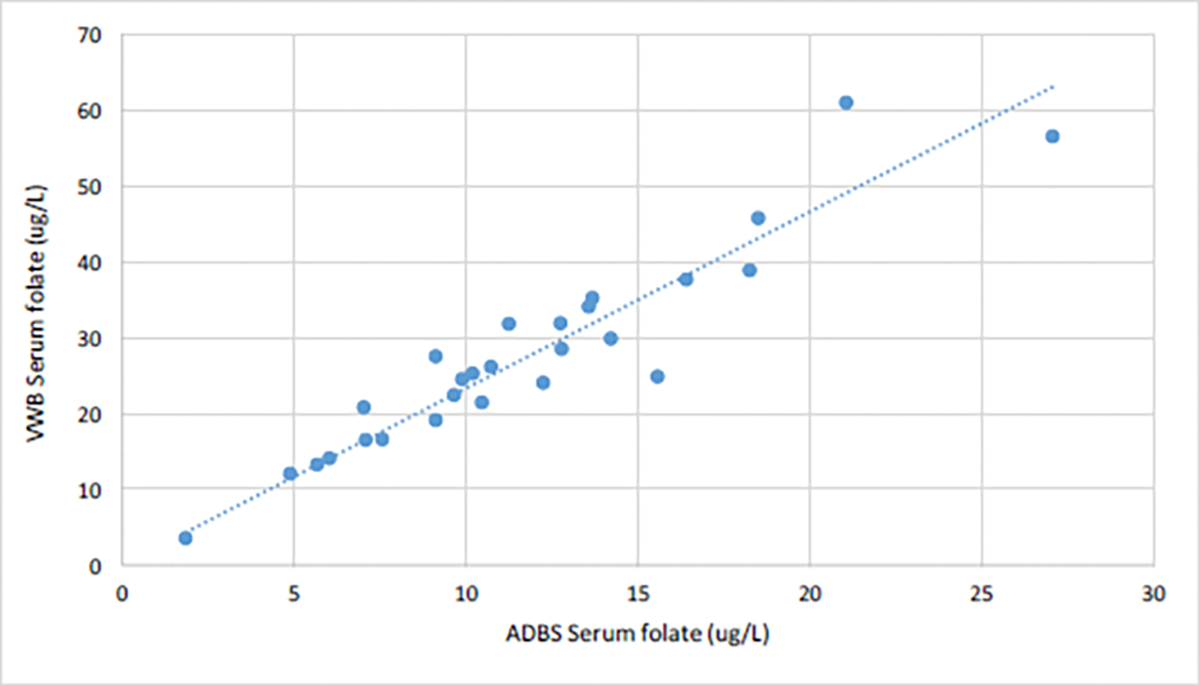
Serum folate values ADBS^1^ and VWBS^2^. **Note:** ^1^Arrayit Dried Blood Spots; ^2^Venous Whole Blood Sample.

We assessed erythrocyte folate values of 15 participant samples collected using ADBS and VWB; they were 80.0% female (n=12), and the mean age of participants was 39.20 (SD=5.69, range=22–68). Erythrocyte folate values from the two methodologies indicate a strong linear relationship, with the two variables accounting for most of the variance in a linear regression model (R^2^=0.83) (Figure 3). We also assessed serum folate values of 26 participant samples collected using ADBS and VWB; they were 80.8% female (n=21), and the mean age of participants was 36.84 (SD=15.54, range=22–65). Serum folate values from the two methodologies indicated a strong linear relationship, with the two variables accounting for most of the variance in a linear regression model (R^2^=0.88) (Figure 4). Linear regression analyses of the two comparisons suggests, that for erythrocyte folate values, VWB=0.9922 × ADBS and for serum folate values, VWB=2.3321 × ADBS.

## Discussion

Arrayit linear flow chromatography dried blood spot cards are useful for assessment of erythrocyte and serum folate. Serum and erythrocyte folate values from other validated methodologies showed a strong linear relationship.

Validation of linear flow chromatography dried blood spot cards for assessment of folate levels in erythrocytes and serum is important because DBS can be collected by non-medically trained personnel in non-medical environments. Moreover, cooperation rates for dried blood spots remain high, even among older populations [18]. Expanded methodologies for assessing folate may allow better testing in hard to reach areas or populations in order to address the global burden of Neural Tube Defects (NTDs), particularly those in developing countries, where a great burden of neonatal death is attributable to NTDs [23].

## Conclusion

This work is critically important because universal supplementation may contribute to the burden of autism spectrum disorders by increasing the proportion of women with folate levels that are too high. In order for women of childbearing potential to fall into the appropriate range for folate status, we must improve access to serum and erythrocyte folate testing. Serum or plasma separation cards such as the Arrayit linear flow chromatography dried blood spots tested in our assays should offer ease of procurement, low blood volume requirement, and reduced exposure to biohazards with linearly related erythrocyte and serum folate values.
